# The relationship between flow experience in sport and subjective well-being among university students: the chain mediation role of psychological resilience and basic psychological needs satisfaction

**DOI:** 10.3389/fpsyg.2026.1818942

**Published:** 2026-05-13

**Authors:** Hongguo Guan, Xiaoyan Li

**Affiliations:** 1 Department of Physical Education and Military Training, Hainan Vocational University of Science and Technology, Haikou, Hainan, China; 2 Department of General Education, Hainan Vocational University of Science and Technology, Haikou, Hainan, China

**Keywords:** basic psychological needs satisfaction, flow experience in sport, psychological resilience, subjective well-being, university students

## Abstract

**Background:**

Subjective well-being is a crucial indicator of university students’ mental health. Sports serve as a key channel for promoting the physical and mental health of university students. Flow experience in sport, as an easily accessible positive psychological experience, is a key approach to enhancing the subjective well-being of university students.

**Methods:**

Grounded in Self-Determination Theory, we built a chain mediation model. Our aim is to examine the chain mediating role of basic psychological needs satisfaction and psychological resilience. This mechanism connects flow experience in sports among university students with their subjective well-being. We collected 488 valid responses by randomly sampling from three universities. We used several scales for measurement. It includes the Flow Experience Scale, the Subjective Well-Being Scale, the Basic Psychological Needs Satisfaction Scale, and the Psychological Resilience Scale. Then, we utilized the PROCESS macro (Model 6, Version 4.0) in SPSS 26.0 to analyze the data and test the chain mediation model.

**Results:**

Among university students, flow experience in sport shows a clear positive link to subjective well-being. Basic psychological needs satisfaction and psychological resilience each played a partial mediating role. And together they form a prominent chain mediation path. Respectively, 12.07%, 13.76%, and 7.77% of the total effect were attributed to these three indirect effects.

**Conclusion:**

This work clarifies the psychological mechanism linking flow experience in sport and subjective well‑being among university students. These research findings also provide practical references for universities. They can implement a comprehensive intervention program that integrates exercise with mental health.

## 1 Introduction

Subjective well-being (SWB) reflects an individual’s perception of their overall quality of life. It also captures their positive emotional experiences. This is a crucial indicator for assessing a person’s mental health and overall quality of life (Diener et al., 1999; Wilson, 1967). Contemporary university students are confronted with various pressures. These include academic competition, interpersonal adaptation, and self-development. Their level of SWB directly links to their psychological state and academic performance. Furthermore, SWB is closely linked to their life development (Yirtici and Duy, 2025). Recent studies have shown that the level of SWB reported by some university students is relatively low. They frequently experience negative emotions. As a result, they find it difficult to effectively deal with various life challenges (Liu et al., 2025). Against this background, exploring ways to improve SWB of university students and analyzing the underlying mechanisms thereof hold significant theoretical and practical value. These efforts are of vital importance in promoting the all-round development of students’ physical and mental health as well as cultivating positive psychological qualities.

Studies have shown that moderate exercise can effectively alleviate the psychological stress of university students and improve their emotional state. Thus, it is positively related to their SWB (Liu et al., 2025). In sports activities, the quality of the exercise experience among university students is a key factor in converting the physical and mental health benefits into an increase in happiness. Among the many factors associated with SWB, flow experience in sport (FES) represents a high-quality positive psychological state. It has increasingly become a focus of research (Duan et al., 2022). FES refers to the optimal mental state that an individual achieves during sport. This state involves complete immersion, the integration of action and consciousness, as well as the balance between skills and challenges (Murphy et al., 2022; Waldman, 1990). In daily life, physical activities are an important way to relax the body and mind as well as for personal development. FES can effectively alleviate psychological stress and help accumulate positive emotions. Previous studies have shown that the experience of flow during exercise is positively linked to greater SWB (Zou et al., 2024). It is worth noting that the development of FES may also relate to characteristics of the exercise environment. Some studies have shown that physical activity in natural settings often brings more pleasant subjective experiences, stronger feelings of relaxation, and greater restorative effects, and is associated with higher SWB (Calogiuri and Chroni, 2014). Research on nature-related experiences has also found that paying attention to everyday natural cues can enhance positive emotions and overall well-being (Gladwell et al., 2013; Passmore and Holder, 2017). In addition, phenomenological research on extreme sports suggests that high levels of concentration, presence, and meaning in specific sport contexts may contribute to the emergence of positive psychological feelings (Brymer and Schweitzer, 2013). These findings suggest that FES may not arise in isolation. Instead, it may be more easily elicited with support from specific environments and may then further relate to positive psychological outcomes. Nevertheless, existing studies have focused more on the psychological benefits of outdoor or nature-based activities themselves. There is still a lack of integrative empirical research on how FES may further relate to college students’ SWB through basic psychological need satisfaction and psychological resilience. Grounded in Self-Determination Theory (SDT), the emergence of positive psychological experiences, such as flow, closely ties to the satisfaction of basic psychological needs (autonomy, competence, and belongingness). Some research suggests that consistently meeting these basic psychological needs can help individuals develop stronger psychological resources, such as psychological resilience (Deci and Ryan, 2000). In fact, psychological resilience and the basic psychological needs satisfaction (BPNS) also play significant roles in enhancing SWB. These factors are the key variables in the psychological growth of university students (Zhang et al., 2026; Zhu et al., 2025). The degree of satisfaction with these demands is directly linked to the expression of positive mental functions (Deci and Ryan, 2000). Psychological resilience is a crucial personal resource for coping with stress and adapting to adversity. It helps university students to buffer against negative impacts and maintain a positive mindset. Psychological resilience is significantly and positively linked to SWB (Puia et al., 2025).

Existing research has separately explored the relationships between FES, BPNS, psychological resilience, and the SWB among university students. However, no research to date has tested their potential chain mediating role within this relationship. We developed a chain mediation model grounded in SDT. We aimed to examine the chain mediating role of BPNS and psychological resilience. And explore the potential mechanism of the correlation between the FES and university students’ SWB. These research findings are intended to provide empirical references for enhancing the SWB of university students.

## 2 Hypotheses

### 2.1 Flow experience in sport and subjective well-being

Flow experience in sport, as the optimal mental state in an athletic environment, enables people to experience profound pleasure and satisfaction during physical activities. The occurrence of FES has been linked to various individual difference factors; for example, research on outdoor athletes indicates that curiosity, exploration, and situational intrinsic motivation serve as significant predictors of recreational flow experience (Emir and Yildiz, 2023). Positive psychological experiences accumulate over time and can have a positive impact on an individual’s overall mental state. It has been continuously receiving attention in the fields of sports psychology and health psychology (Murphy et al., 2022; Zou et al., 2024). In recent years, mounting empirical evidence has consistently indicated that FES is significantly and positively linked to SWB among university students. This correlation has been widely validated (Duan et al., 2022; Liao et al., 2023). Ding et al. (2023) carried out a cross-sectional survey of 350 university students. The results showed that students with higher levels of FES reported higher SWB scores. Similarly, Zou et al. (2024) also shows that FES is significantly and positively linked to SWB. This suggests that individuals who frequently experience flow during physical activity often report enhanced feelings of life meaning and engagement. Their SWB also improves accordingly (Bayram et al., 2025). Based on these studies, we propose the following hypothesis:

*H1*: FES demonstrated a positive association with SWB.

### 2.2 The mediating role of basic psychological needs satisfaction

The BPNS originates from SDT. It refers to the feeling of a person when their three needs (i.e., autonomy, competence and relatedness) are fully met. The degree of satisfaction of these demands serves as the fundamental basis for stimulating intrinsic motivation, promoting positive psychological adjustment, and integrating one’s personality (Deci and Ryan, 2000). The sports environment provides a unique setting to BPNS. FES, the optimal state, requires a high degree of concentration as well as a balance between skills and challenges. The process of achieving this state may itself be related to these three basic needs. First of all, individuals’ independent choices and deep engagement in activities will enhance their sense of autonomy. Second, successfully meeting athletic challenges provides immediate competence feedback, boosting their sense of competence. Finally, in team sports or shared athletic environments, coordinated teamwork and emotional connection with others fulfill their sense of relatedness (Domingues et al., 2023). Existing research indicates that FES, as a typical optimal experience, positively correlates with the level of BPNS (Sim et al., 2025). University students who show higher engagement and immersion in sport often report stronger perceived control over their exercise behavior, stronger competence when their skills match challenges, and higher levels of belonging in sports settings (Liao et al., 2023). At the same time, several empirical studies have validated the link between BPNS and SWB in university students (Leow et al., 2023; Zhou et al., 2025). Zhang et al. (2024) conducted a study with 835 university students. Their research revealed a significant positive link from BPNS to SWB. Furthermore, it verified that BPNS mediated the association between sports anxiety and SWB. Overall, there is a close connection between FES and BPNS, and BPNS is correlated with university students’ SWB. We propose the following hypotheses based on previous research:

*H2*: FES is positively associated with BPNS.

*H3*: BPNS is positively associated with SWB among university students.

*H4*: BPNS mediates the link between FES and SWB among university students.

### 2.3 The mediating role of psychological resilience

Psychological resilience is a core construct in positive psychology. It describes the dynamic psychological ability of an individual to flexibly mobilize internal psychological resources when facing adversity, stress, or trauma. This ability helps them maintain stable psychological functions, including positive adaptation and psychological recovery (Qin et al., 2023; Wang et al., 2025). Among university students, psychological resilience serves as a crucial psychological support for coping with academic pressure, interpersonal challenges, and life changes. It is tightly connected with engaging in physical exercise and maintaining a positive mental state. This makes it a key topic in sports psychology and research on the mental health of university students (Karababa et al., 2025; Liu et al., 2024). Studies have shown that positive experiences in a sports environment, such as flow experience, are positively linked to the development of an individual’s psychological resilience (Li and Pan, 2025). When people experience the state of flow during exercise, the sense of immersion and control brought by the task may be associated with a stronger sense of self-efficacy and psychological resilience. It is worth noting that this higher level of psychological resilience is also significantly positively correlated with a person’s SWB (Puia et al., 2025). People with strong psychological resilience tend to report higher SWB when facing daily challenges (Reyes-Perez et al., 2025). Grounded in SDT, BPNS is a crucial environmental condition for cultivating and establishing individual psychological resilience, and psychological resilience is a core psychological resource (Deci and Ryan, 2000). In the specific context of sport, flow experience typically arises from autonomous participation. These features essentially reflect psychological needs satisfaction. This state that meets such needs may provide important conditions for an individual to develop psychological resilience. In line with this reasoning, we suggest that psychological resilience plays a crucial role in linking FES to SWB. On this basis, we hypothesize that:

*H5*: FES is positively related to psychological resilience.

*H6*: Psychological resilience is positively correlated with SWB among university students.

*H7*: Psychological resilience mediates the link of FES to SWB in university students.

### 2.4 The chain mediating role of basic psychological needs satisfaction and psychological resilience

Research has shown that BPNS positively correlates with psychological resilience. Specifically, greater BPNS is typically linked to stronger psychological resilience. It is also accompanied by more effective stress management methods and psychological adaptation outcomes (Bai and Bai, 2024). This association suggests that BPNS serves as a basic condition for psychological development. As a crucial internal process, it links positive experiences with the long-term accumulation of psychological resources (Song and Yan, 2025). When an individual’s needs for autonomy, competence and relationships are fully met, they tend to possess more abundant internal resources. These resources help individuals develop adaptive coping strategies, maintain emotional stability. This state, consequently, corresponds to greater psychological resilience (Sánchez, 2022). SDT offers a clear explanatory framework. This framework helps to explain the association of FES with SWB among university students. It also facilitates our exploration of the chain mediating roles of BPNS and psychological resilience (Deci and Ryan, 2000). This view suggests that FES, as the optimal psychological state an individual reaches during a specific activity, often overlaps with the conditions of BPNS (Sim et al., 2025). In addition, theoretically, the continuous BPNS is a vital foundation for the development of stable psychological resources. The accumulation of these resources is often associated with increases in psychological resilience (Bai and Bai, 2024). As a fundamental psychological asset, psychological resilience is positively linked to greater SWB (Pan et al., 2025). In summary, a direct association exists between FES and the SWB of university students. FES may also show systematic connections with BPNS and psychological resilience. We therefore hypothesize that:

*H8*: BPNS and psychological resilience mediate the link of FES to SWB among university students.

**Figure 1:**
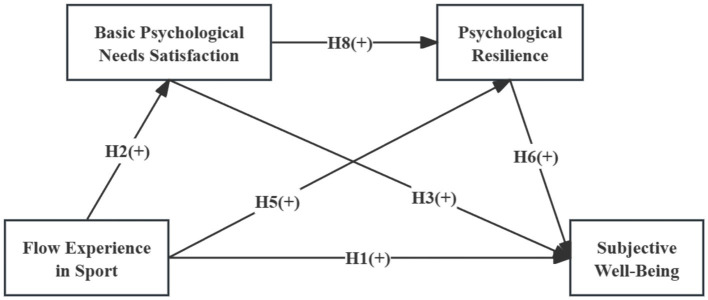
Hypothesized research model.

The work draws on SDT. FES, BPNS, psychological resilience, and SWB in university students may form a sequential pathway. This pathway starts from positive situational experience, goes through the satisfaction of core motivational mechanisms and the accumulation of stable psychological resources, and finally leads to overall well-being. To systematically test this mechanism, we have constructed a theoretical model, as illustrated in Figure 1.

## 3 Methods

### 3.1 Sample and data collection procedure

We collected data through the online survey platform Questionnaire Star^1^. We then tested the proposed hypothesized model. Using convenience sampling, we recruited university students from three universities located in Hainan Province, to increase the diversity of the sample. The participating universities included HAINAN NORMAL UNIVERSITY, QIONGTAI NORMAL UNIVERSITY and HAIKOU UNIVERSITY OF ECONOMICS. The students were enrolled in various academic majors, including Sports, Education, Psychology and Management. We collected data from December 2025 to January 2026. Moreover, every participant in this research engaged in the work voluntarily. They provided explicit consent for the protection of their private information and data privacy. We clearly promised to protect their personal information. The inclusion criteria were as follows: being a currently enrolled undergraduate student, being aged 18 years or above, and agreeing to participate voluntarily. The exclusion criteria were as follows: incomplete questionnaires, patterned or repetitive responses across all items, and responses that failed the attention check, if applicable (Schroeders et al., 2022). In addition, participants who started but did not complete the survey were treated as dropouts and were not included in the final analysis. A total of 540 university students were invited to participate. After data screening and based on the pre-defined exclusion criteria, 52 questionnaires were excluded. The final sample consisted of 488 valid questionnaires. Based on the collected questionnaires, the effective response rate was 90.37%. Table 1 displays the detailed background characteristics of the people. Three hundred thirteen were male (64.1%) and 175 were female (35.9%). In terms of age, most participants fell within the 18 to 20 range (303 individuals, 62.1%). The majority of participants were freshmen, with 235 students (48.2%). Overall, the sample shows a reasonable distribution across key demographic variables. It has good representativeness and meets the requirements for further analyses. Overall, the sample covered students from multiple universities, provinces, and academic majors, providing an adequate basis for the subsequent analyses.

**Table 1:** Descriptive statistics.

Participant demographics	Variable	Quantity	Percentage
Gender	Male	313	64.1%
	Female	175	35.9%
Age	18–20 years	303	62.1%
	21–22 years	151	30.9%
	Over 22 years old	34	7.0%
Grade	Freshmen	235	48.2%
	Sophomores	144	29.5%
	Juniors	92	18.8%
	Seniors	17	3.5%

### 3.2 Measures

The questionnaire had two sections. The initial section collected the demographic information of the participants. In the following section, we cover the key variables examined in this work. In this study, we used the established scales to assess all the constructs. We adapted these scales appropriately to fit the study’s context and objectives.

We adapted the FES Scale from the Flow Short Scale (Engeser and Rheinberg, 2008). The scale includes 10 items across two dimensions: performance fluency (6 items) and activity absorption (4 items) (Tian et al., 2022). We rated the items using a seven-point Likert scale. For example: “During exercise, I become fully absorbed in what I am doing”. Its Cronbach’s *α* coefficient is 0.911.

We adopted the BPNS Scale developed by Sheldon and Niemiec (2006). The scale covers three needs: autonomy, competence, and relatedness. It contains nine items in total, with three items for each dimension (e.g., “In my life, I have successfully completed difficult tasks and plans”). We used a 7-point scoring system. Higher scores reflect greater BPNS (Xu et al., 2025). The scale yielded a Cronbach’s *α* of 0.905.

We assessed psychological resilience among university students with the scale developed by Campbell-Sills and Stein (2007). The scale has 10 items. Each item uses a 5-point scale. Higher total scores reflect greater psychological resilience (e.g., “When facing problems, I see the humorous or positive side”) (Yang et al., 2025). Its Cronbach’s *α* coefficient is 0.902.

We measured SWB with the instrument created by Diener et al. (1985), with a total of 5 questions (e.g., “I am very satisfied with my life”). The scale shows strong applicability in Chinese research contexts (Pan et al., 2022). We used a 7-point Likert format for this scale (Lin, 2023). Its Cronbach’s *α* coefficient is 0.826.

### 3.3 Data analysis

The work used the bootstrap method (Model 6) from the SPSS PROCESS macro to conduct mediation analysis. FES was the independent variable, with BPNS and psychological resilience as mediators, and SWB as the dependent variable. We included demographic factors such as gender, age, and grade as covariates. We set the statistical significance of mediating variables at a 95% confidence interval (CI) and used 5,000 bootstrap samples (Preacher and Hayes, 2008). We considered the indirect effect significant when the 95% CI from the bootstrap method did not include zero (Hayes and Rockwood, 2017).

## 4 Results

### 4.1 Measurement instrument

According to the suggestions of Hair et al. (2021), we evaluated the reliability and validity of the scale through model analysis. Item outer loadings and composite reliability were adopted as the key reliability indices. Per the guidelines, item outer loadings must exceed 0.708 (Hair et al., 2021). Table 2 presents the results, all items demonstrated satisfactory outer loadings, and all constructs showed high composite reliability.

**Table 2:** Reliability and validity.

Constructs	Items	Loadings	CR	Cronbach’s α	AVE
FES	FES1	0.718	0.926	0.911	0.556
	FES2	0.706			
	FES3	0.724			
	FES4	0.764			
	FES5	0.749			
	FES6	0.753			
	FES7	0.778			
	FES8	0.754			
	FES9	0.758			
	FES10	0.749			
BPNS	BPNS1	0.732	0.922	0.905	0.569
	BPNS2	0.779			
	BPNS3	0.766			
	BPNS4	0.782			
	BPNS5	0.745			
	BPNS6	0.746			
	BPNS7	0.754			
	BPNS8	0.740			
	BPNS9	0.744			
Psychological resilience	Psychological Resilience1	0.749	0.919	0.902	0.531
	Psychological Resilience2	0.741			
	Psychological Resilience3	0.711			
	Psychological Resilience4	0.723			
	Psychological Resilience5	0.735			
	Psychological Resilience6	0.702			
	Psychological Resilience7	0.732			
	Psychological Resilience8	0.728			
	Psychological Resilience9	0.743			
	Psychological Resilience10	0.723			
SWB	SWB1	0.770	0.877	0.826	0.589
	SWB2	0.783			
	SWB3	0.780			
	SWB4	0.764			
	SWB5	0.739			

FES, flow experience in sport; BPNS, basic psychological needs satisfaction; SWB, subjective well-being; CR, composite reliability; AVE, average variance extracted.

We then assessed validity by examining convergent and discriminant validity. We set the average variance extracted (AVE) threshold above 0.50 to evaluate convergent validity (Hair et al., 2021). Discriminant validity was evaluated through both the heterotrait–monotrait ratio of correlations (HTMT) and the Fornell-Larcker criterion. All HTMT values were below the suggested cutoff of 0.850 (see Table 3) (Hair et al., 2021). The correlations between each pair of constructs did not exceed the square root of their respective AVE (see Table 4), they meet the Fornell-Larcker criterion (Hair et al., 2021). These results, from both the HTMT and Fornell–Larcker approaches, indicate adequate discriminant validity for all constructs.

**Table 3:** Discriminant validity (HTMT standard).

Variable	SWB	BPNS	Psychological Resilience	FES
SWB				
BPNS	0.596			
Psychological Resilience	0.653	0.666		
FES	0.739	0.650	0.673	

FES, flow experience in sport; BPNS, basic psychological needs satisfaction; SWB, subjective well-being. For discriminant validity to be established, all values must be maintained under the cutoff of 0.850.

**Table 4:** Fornell–Larcker criteria.

Variable	SWB	BPNS	Psychological resilience	FES
SWB	***0.767***			
BPNS	0.526	***0.754***		
Psychological Resilience	0.574	0.604	***0.729***	
FES	0.652	0.595	0.615	***0.746***

FES, flow experience in sport; BPNS, basic psychological needs satisfaction; SWB, subjective well-being. The Fornell–Larcker criterion stipulates that for discriminant validity to hold, each construct’s correlation with others must not exceed the square root of its own AVE (shown in bold italics on the diagonal).

### 4.2 Common method bias

We adopted one approach to evaluate common method bias (CMB). Harman’s single-factor test showed that no single factor accounted for most of the variance (Podsakoff et al., 2003). Only 39.101% of the variance was explained by the largest single factor. This value fell far below the 50% threshold (Podsakoff et al., 2003). Therefore, based on these results, we infer that the data did not show substantial CMB.

### 4.3 Collinearity assessment

We assessed multicollinearity in the model by examining the variance inflation factor (VIF) values of the latent variables (Hair et al., 2021). All VIF scores for the latent constructs were below 3.3, which is the recommended cutoff. This shows no multicollinearity problem in the result (Hair et al., 2021). All VIF values in Table 5 fall below the 3.3 cutoff. Multicollinearity does not bias the work’s findings.

**Table 5:** Variance inflation factor (VIF).

Variable	SWB	BPNS	Psychological resilience	FES
SWB				
BPNS	1.803		1.547	
Psychological resilience	1.874			
FES	1.841	1.000	1.547	

FES, flow experience in sport; BPNS, basic psychological needs satisfaction; SWB, subjective well-being.

### 4.4 Descriptive statistics and correlation analysis

We conducted descriptive statistics and Pearson correlation analyses for the key research variables. This helped us understand their basic distribution characteristics and the relationships among variables. As shown in Table 6, for all variables, the absolute values of skewness were below 2, and the absolute value of kurtosis is also below 7. This demonstrates that the data were approximately normally distributed. The correlation results show that FES was significantly and positively linked to BPNS, psychological resilience, and SWB.

**Table 6:** Correlation analysis (*N* = 488).

Constructs	*M* ± SD	SK	Kur	FES	BPNS	Psychological resilience	SWB
FES	4.55 ± 1.00	−0.949	0.681	1			
BPNS	4.74 ± 0.95	−0.979	0.574	0.589***	1		
Psychological resilience	3.46 ± 0.59	−1.316	1.639	0.608***	0.602***	1	
SWB	4.73 ± 1.17	−0.875	0.189	0.636***	0.512***	0.559***	1

***p < 0.001; FES, flow experience in sport; BPNS, basic psychological needs satisfaction; SWB, subjective well-being.

### 4.5 Confirmatory factor analysis

To compare the scale’s structure to our theoretical model, we used CFA. A strong model fit was shown by the results. All fit measures met recommended thresholds (Ji et al., 2024). See Table 7 for details.

**Table 7:** Model fit indices.

Fit index	Reference value	Final model
CMIN/DF	<5	1.609
RMSEA	<0.08	0.035
TLI	>0.9	0.959
CFI	>0.9	0.961
IFI	>0.9	0.959

CMIN, chi-square value; DF, degrees of freedom; RMSEA, root mean square error of approximation; CFI, comparative fit index; IFI, incremental fit index; TLI, Tucker–Lewi’s index.

### 4.6 Mediating effect analysis

We ran the PROCESS macro (Model 6) in SPSS Statistics 26 (Hayes, 2017). We used the bias-corrected percentile Bootstrap method with 5,000 resamples. We standardized all variables and included three covariates: gender, age, and grade. We tested the chain mediating roles of BPNS and psychological resilience in the association of FES with SWB. The results, shown in Table 8, indicate that FES relates positively and significantly to BPNS (*β* = 0.540, *p* < 0.001), psychological resilience (*β* = 0.381, *p* < 0.001), and SWB (*β* = 0.220, *p* < 0.001). BPNS shows a notable positive correlation with psychological resilience (*β* = 0.399, *p* < 0.001) and SWB (*β* = 0.074, *p* < 0.01). Psychological resilience is significantly and positively linked to SWB (*β* = 0.120, *p* < 0.001).

**Table 8:** Regression results for the mediation model.

Result variable	Predictor variable	*β*	SE	*T*	Bootstrap 95% CI		*R* ^2^	*F*
					LLCI	ULCI		
BPNS	FES	0.540	0.033	16.176***	0.474	0.6056	0.355	66.440
	Gender	0.513	0.522	0.982	−0.513	1.540		
	Age	−0.406	0.508	−0.799	−1.404	0.592		
	Grade	−0.303	0.361	−0.837	−1.013	0.408		
Psychological resilience	FES	0.381	0.041	9.3734***	0.301	0.461	0.462	82.825
	BPNS	0.399	0.045	8.924***	0.311	0.486		
	Gender	−0.245	0.513	−0.478	−1.254	0.763		
	Age	−0.056	0.499	−0.111	−1.036	0.925		
	Grade	−0.047	0.355	−0.133	−0.745	0.650		
SWB	FES	0.220	0.023	9.519***	0.175	0.266	0.471	71.502
	BPNS	0.074	0.025	2.942***	0.025	0.124		
	Psychological Resilience	0.120	0.024	5.021***	0.073	0.166		
	Gender	0.077	0.268	0.285	−0.451	0.604		
	Age	−0.014	0.261	−0.053	−0.526	0.499		
	Grade	0.277	0.186	1.493	−0.088	0.642		

FES, flow experience in sport; BPNS, basic psychological needs satisfaction; SWB, subjective well-being.

**Figure 2:**
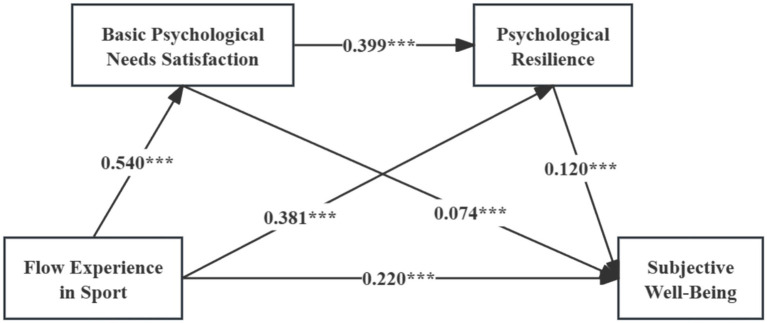
Mediation model. ***p < 0.001.

The mediation effects (displayed in Table 9 and Figure 2) demonstrate that this study examined both the direct and total effects of FES on SWB, along with the indirect effects of BPNS and psychological resilience. Our results showed that BPNS and psychological resilience each partially mediated the link from FES to SWB. In addition, the two variables served as a chain mediator between FES and SWB. Therefore, H4, H7, and H8 are supported.

**Table 9:** Mediation analysis.

Effect	Path	*β*	Percentage	95%CI		Type of mediation
				LLCI	ULCI	
Total effect	FES → SWB	0.331	100%	0.296	0.367	Partial mediation
Direct effect	FES → SWB	0.220	66.39%	0.175	0.266	
Indirect effect	FES → BPNS → SWB	0.040	12.07%	0.010	0.075	
	FES → Psychological Resilience →SWB	0.046	13.76%	0.022	0.071	
	FES → BPNS → Psychological Resilience → SWB	0.026	7.77%	0.012	0.041	

FES, flow experience in sport; BPNS, basic psychological needs satisfaction; SWB, subjective well-being.

## 5 Discussion

We aimed to investigate how FES relates to SWB among university students, drawing on SDT. We further verified the chain mediating role of BPNS and psychological resilience. It is hypothesized that FES is positively linked to SWB in university students, with BPNS and psychological resilience mediating the association of FES with SWB. We discuss the key results corresponding to our research questions and hypotheses. These discussions are presented in detail in the following sections.

This work shows a significant positive correlation. This correlation exists from FES to SWB among university students. Therefore, H1 is supported. This result is consistent with Ding et al. (2023). On the one hand, exercise can lead to changes in the structure and function of an individual’s brain. These changes can boost positive emotions and ease symptoms of depression and anxiety. Therefore, they correspond to greater SWB. As a high-quality form of exercise, FES can enhance these benefits (Shang et al., 2021). University students who frequently experience the flow state during physical activities tend to gain deep enjoyment and self-affirmation from it. This positive experience can effectively alleviate the psychological stress caused by academic and interpersonal pressures. The results show that people with greater life satisfaction also experience more positive emotions (Liao et al., 2023). When people enter the state of flow during sports activities, their attention will be completely absorbed in the activity itself. They will temporarily escape from external evaluation pressures and distractions from daily life. This state of total concentration and uninterrupted immersion can effectively alleviate mental fatigue. It can help university students achieve a brief mental relaxation and inner satisfaction (Huang W. Z. et al., 2025). This indicates that frequent FES can bring about immediate positive emotions. They also create conditions for maintaining long-term psychological stability and SWB. Overall, this highlights the significant role of FES in promoting the emotional health of university students and fostering positive psychological qualities.

Our further analysis revealed that BPNS plays a vital mediating role in the link of FES with SWB. Therefore, H2, H3, H4 were supported. These results support the conclusions of Sim et al. (2025) and Zhang et al. (2024). SDT posits that, the satisfaction of autonomy, competence and belongingness is a crucial internal condition for individuals to promote mental health and enhance happiness (Ryan and Deci, 2017). FES represents an ideal state of positive psychology. It provides an essential contextual foundation for fulfilling these three fundamental psychological needs. The core characteristics of the flow experience are the matching of skills and challenges, as well as the integration of action and consciousness. This group of university students experienced a greater sense of autonomy and control. They can more clearly observe the improvement of their skills as well as their increased autonomy and competence. Moreover, it often shows a positive correlation with BPNS (Liao et al., 2023). University students with a high degree of BPNS exhibit more positive self-acceptance and have more optimistic expectations for life. This psychological state shows a highly consistent correlation with SWB (Leow et al., 2023). These results suggest that universities can use BPNS as a central leverage point to strengthen the psychological well-being of university students. Universities can both engage students in diverse sports activities to create favorable conditions for achieving flow, and implement support mechanisms oriented toward psychological needs.

Psychological resilience significantly mediated the association of FES with SWB, confirming H5, H6, and H7. This finding aligns with the findings reported by Li and Pan (2025) and Puia et al. (2025). For university students with higher FES, they will experience stronger feelings of control and greater accomplishment. This will accumulate internal resources for coping with challenges, which is directly related to psychological resilience. Li and Pan (2025) also found a positive link between FES and psychological resilience. Studies have also shown that psychological resilience relates positively to the SWB of university students (Dalmis et al., 2025). University students possessing greater psychological resilience more readily discover their own value in academic, interpersonal, and other life situations, and to set clear life goals. This clear understanding and active pursuit of life purpose closely links to high SWB (Xiang, 2024). Therefore, this study proposes a feasible intervention strategy. We can enhance the FES of university students and cultivate their psychological resilience to elevate their SWB.

We also discovered the chain mediating role of BPNS and psychological resilience. They linked FES to the SWB of university students, confirming Hypothesis 8. We found that when university students engage in physical activities and experience a state of flow, they are more likely to view their mental state in a positive and accepting manner. Then, they tended to maintain a more stable and sufficient level of BPNS. Grounded in SDT, the perception of positive psychological experiences is linked to the accumulation of personal mental resources (Deci and Ryan, 2000). When university students receive sufficient support in terms of autonomy, ability and sense of belonging, they will develop a more positive self-perception. They can also propose more flexible strategies for solving problems. Moreover, they may also establish a more stable social support network. These factors all help them to deal with stress and challenges with greater confidence and stronger adaptability. This is related to a greater degree of psychological resilience (Bai and Bai, 2024; Huang C. et al., 2025). It helps student buffer against life stress, assists in regulating negative emotions, and enables them to accumulate positive emotions and enhance their self-identity. This is often associated with SWB (Zhou and Chen, 2024). Therefore, the chain mediation pathway in this study reveals a positive transformation process. It demonstrates the correlation between FES and BPNS. These findings have sharpened our insight into the link from FES to SWB and the underlying mechanisms involved. They also provide a practical and feasible entry point for enhancing the SWB among university students.

## 6 Implications and limitations

### 6.1 Theoretical implications

This work indicates a clear positive association of FES with the SWB among university students. It also confirmed the chain mediated mechanism of BPNS and psychological resilience within it. These research findings have further enriched and deepened the theoretical understanding of the formation mechanism of SWB among university students. This study extends the application of SDT to FES research. This indicates that this theory can provide an effective explanation for these associations. It accounts for the links among external positive experiences, satisfaction of psychological needs, accumulation of psychological resources, and positive psychological outcomes. This has expanded the application scope of this theory in positive psychology and sports psychology. It also provides theoretical support and research ideas for further discussions in related fields regarding the connection between external positive experiences and individual positive psychological development.

### 6.2 Practical implications

We identified a clear positive link from FES to SWB among university students. This discovery provides specific and practical directions for intervention measures aimed at promoting the positive mental health of this group of people. For individuals, they can actively protect and build positive psychological resources. They can do this through small, practical adjustments in their daily physical activities. We believe that during the exercise period, a highly focused environment can be created to eliminate external distractions. Pay attention to your body sensations and the details of your movements. This can help deepen the FES. For example, runners can set their phones to silent mode. They can focus on their breathing and footsteps, and not think about anything else. Furthermore, during exercise, one can think about how to solve the problems they encounter. Over time, this will enhance one’s mental strength. It makes you better at handling stress. It enables you to be better at coping with stress, improve your ability to manage emotions. It creates a solid base for better SWB.

Several results from this research indicate that campus environment and course support matter considerably for positive mental growth in university students. For the university sports teaching department and the student management department, they can optimize the course design and activity organization, providing practical support for students’ flow experience and psychological resource accumulation. First, our findings show BPNS correlates positively with student well-being. Therefore, we suggest that we could design classroom interaction and teamwork activities, enhance peer support and teacher encouragement, and establish a friendly sports exchange platform. This can enhance students’ sense of autonomy, competence and belongingness in physical activities. They help fully meet students’ basic psychological needs. Second, our results show psychological resilience correlates positively with SWB. So, sports clubs, class activities and campus competitions can be utilized to organize regular and interesting sports events. These activities should be low-pressure, highly participatory, and focus on the process. They do not need complex planning or high cost. Furthermore, mental health education can be appropriately integrated with physical education. For instance, during the final exam period, short lectures or group activities on psychological resilience can be provided. This helps students to scientifically understand the dual benefits of exercise in terms of emotional regulation and psychological development. These approaches may help make physical activity a routine, low-cost method for university students to maintain their mental health and enhance their SWB.

### 6.3 Limitations and directions for future research

This work preliminarily reveals the association of FES with SWB among university students and systematically explores the chain mediation role of BPNS and psychological resilience. But this work has some limitations. These shortcomings also highlight possible avenues for subsequent studies.

We adopted a cross-sectional design. All data were collected at a single time point to examine the links among the variables. This is unable to capture the dynamic development process and long-term relationship characteristics among the variables. Additionally, the cross-sectional nature of the study restricts the ability to draw causal inferences. Future research can adopt a longitudinal design. This enables a systematic study of the long-term dynamic relationships among variables. Researchers can also employ statistical methods such as cross-lagged panel analysis. These methods can further clarify the causal direction between variables. And enhance the causal relationship and stability of the research results.

This work is entirely based on the self-reporting measurement method of the participants. All the measurement data were provided by the participants themselves. This might introduce biases such as social expectation deviations or self-perception distortions. This could potentially undermine the objectivity and accuracy of the research conclusions. They may also overstate the estimated correlation between the variables. Future research can incorporate multiple data sources and assessment methods. For instance, teacher or peer evaluations, as well as behavioral observations, can all be utilized to supplement these data. Integrating multiple sources of data will enhance the reliability of the measurement and the robustness of the conclusions.

In our study, only BPNS and psychological resilience were included as sequential mediating constructs in the model, other potential mediating or moderating factors were not considered. For example, factors such as the type of exercise, the duration of exercise, and academic pressure might play a role in it. Future research can expand the boundaries of this model. Researchers can incorporate more background factors and individual factors into the analytical framework. This will help to study their potential regulatory roles or parallel mediating roles. This method enables a fuller and nuanced insight into the complex psychological mechanisms linking FES to university students’ SWB.

## 7 Conclusion

This work explores the link from FES to the SWB of university students. Furthermore, it examines the chain mediating roles of BPNS and psychological resilience in this relationship. We collected self-reported data from 488 university students. Using these data, we conducted an empirical test of the proposed research hypotheses. The results show that FES is significantly positively correlated with SWB, and that BPNS and psychological resilience play a significant chain mediation role between FES and SWB. All of our hypotheses have been verified. This study helps us understand the underlying mechanism linking FES to SWB of university students. For university students, it also offers empirical backing for strengthening their SWB. It also provides empirical evidence for enhancing the psychological well-being of university students from the perspectives of FES, BPNS, and cultivation of psychological resources.

## Data Availability

The raw data supporting the conclusions of this article will be made available by the authors, without undue reservation.

## Footnotes

1
www.Sojump.com
